# The Role of Lutheran/Basal Cell Adhesion Molecule in Hematological Diseases and Tumors

**DOI:** 10.3390/ijms25137268

**Published:** 2024-07-02

**Authors:** Juan Jin, Qinqin Guo, Zhibin Yan

**Affiliations:** College of Life Sciences and Medicine, Zhejiang Sci-Tech University, Hangzhou 310018, China; 20210049@zstu.edu.cn (J.J.); 202230903176@mails.zstu.edu.cn (Q.G.)

**Keywords:** Lu/BCAM, cell adhesion, hematological diseases, tumors

## Abstract

Cell adhesion is a dynamic process that plays a fundamental role in cell proliferation, maintenance, differentiation, and migration. Basal cell adhesion molecule (BCAM), also known as Lutheran (Lu), belongs to the immunoglobulin superfamily of cell adhesion molecules. Lu/BCAM, which is widely expressed in red blood cells, endothelial cells, smooth muscle cells and epithelial cells across various tissues, playing a crucial role in many cellular processes, including cell adhesion, cell motility and cell migration. Moreover, Lu/BCAM, dysregulated in many diseases, such as blood diseases and various types of cancer, may act as a biomarker and target for the treatment of these diseases. This review explores the significance of Lu/BCAM in cell adhesion and its potential as a novel target for treating hematological diseases and tumors.

## 1. Introduction

Cell adhesion molecules (CAMs) are membrane surface proteins that facilitate interactions between cells and the extracellular matrix, as well as between different cells. They can be categorized into selectins, integrins, cadherins, the immunoglobulin superfamily and some other unclassified adhesive molecules based on their structural characteristics. Through specific binding between ligands and receptors, CAMs regulate various cellular functions, such as cell adhesion, cell proliferation, cell apoptosis, cell differentiation, cell motility and cell migration, playing crucial roles in diverse physiological and pathological processes, including embryonic development, tissue differentiation, inflammatory and immune responses, as well as tumor invasion and metastasis [1,2,3]. In normal tissues, the expression of CAMs is precisely controlled. However, aberrant expression can disrupt cellular connections, leading to the onset and progression of various diseases.

Basal cell adhesion molecule (BCAM), also known as the Lutheran blood group glycoprotein (Lu), is a transmembrane glycoprotein belonging to the immunoglobulin superfamily. Originally identified in blood cells, it has since been found to be widely present in endothelial cells, smooth muscle cells and epithelial cells across different tissues. Lu/BCAM functions as a receptor for laminin, an extracellular matrix protein; plays an important role in regulating cell adhesion, motility, migration and invasion; and is involved in hematological diseases and tumors. Abnormal expression of Lu/BCAM has been implicated in the development of various blood disorders and tumors, highlighting its potential as a molecular marker for disease diagnosis and as a therapeutic target.

**Figure 1:**
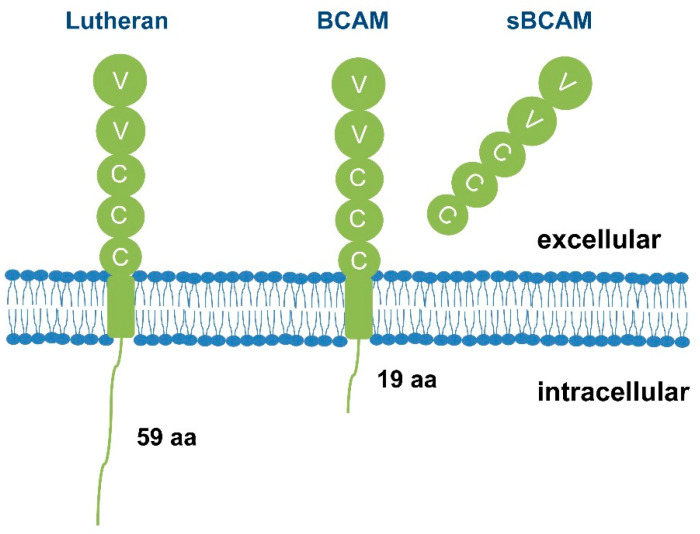
Structural schematic diagram of Lu/BCAM. Lu/BCAM is a transmembrane glycoprotein with a type I single-pass structure. It comprises an extracellular domain, a transmembrane domain, and an intracellular domain. The extracellular domain of Lu/BCAM consists of five immunoglobulin superfamily (IgSF) domains, which include two variable-type domains (V) and three constant-type domains (C). The intracellular domain of the Lutheran glycoprotein spans 59 amino acids, while BCAM has a shorter intracellular domain, consisting of only 19 amino acids. Additionally, Lu/BCAM can be cleaved by MT1-MMP on the cell membrane, leading to the formation of a soluble protein (sBCAM).

The Lu/BCAM gene is located on chromosome 19q13.2 and consists of 15 exons and 14 introns, spanning approximately 12.5 kb. It is expressed in two isoforms, BCAM and Lu, which have 588 and 628 amino acids, respectively [4]. Their structure differs only in the length of the cytoplasmic tail. BCAM is a member of the immunoglobulin superfamily and is primarily expressed in the basal part of epithelial cells. It was initially identified in the human colon cancer cell line HT19 [5]. Lu, as polymorphisms in this gene define the Lu^a^/Lu^b^ Lutheran blood groups, also known as CD239, was later discovered as a homologous isomer of BCAM [6]. Lu/BCAM is a single transmembrane glycoprotein with extracellular, transmembrane and intracellular domains (Figure 1). The extracellular domain consists of five immunoglobulin superfamily (IgSF) domains, including two variable-type domains and three constant-type domains [7]. The intracellular region of the Lu glycoprotein has 59 amino acids, while the intracellular region of BCAM is shorter with only 19 amino acids due to alternative splicing during transcription [8]. The intracellular region of the Lu glycoprotein contains SH3 binding sites and five potential phosphorylation sites, suggesting its involvement in cell signaling transduction [9]. Additionally, Lu/BCAM can be cleaved by membrane type 1-matrix metalloproteinase (MT1-MMP) on the cell membrane, leading to the formation of a soluble protein (sBCAM). It has been found that sBCAM contributes to tumor metastasis [10,11].

Lu/BCAM is widely expressed in hematopoietic, vascular endothelial and epithelial cells across various tissues, including the human brain, liver, colon, placenta and kidneys [9,12]. It belongs to the immunoglobulin superfamily of cell adhesion molecules. The extracellular domain of Lu/BCAM can interact with laminin, integrin or other ligands, facilitating various processes such as cell adhesion, motility, migration and invasion. Its intracellular region can interact with cytoskeletal proteins, such as hemoglobin, to mediate cellular signal transduction. Abnormal expression of Lu/BCAM is closely associated with the occurrence and progression of various diseases, including sickle cell anemia (SCA), liver cancer, lung cancer, colorectal cancer, bladder cancer, etc. [12,13,14,15,16].

## 2. The Role of Lu/BCAM

**Figure 2:**
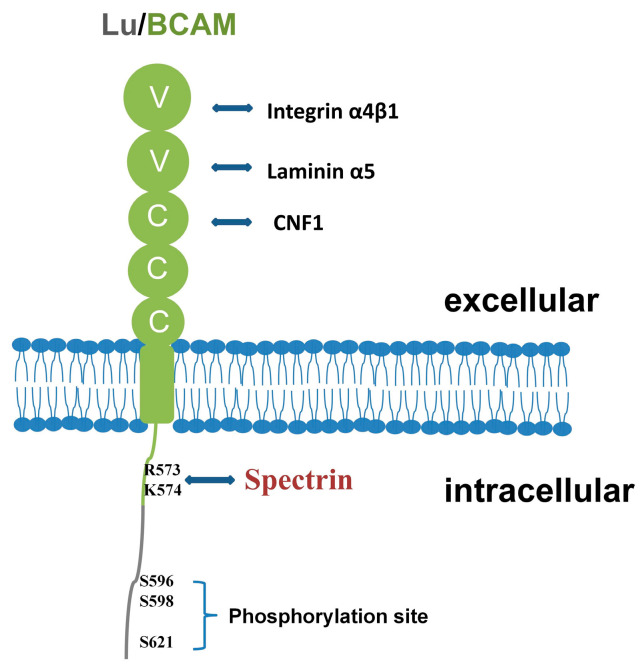
Functional schematic diagram of Lu/BCAM. The extracellular domain of Lu/BCAM can interact with laminin and integrin. It can also bind to cytotoxin necrosis factor CNF1. And the intracellular region of Lu/BCAM can interact with spectrin.

Lu/BCAM, a transmembrane glycoprotein, plays a crucial role in cell adhesion, motility, migration and invasion. Its extracellular domain enables binding to extracellular matrix proteins, such as laminin, integrin and other ligands. Meanwhile, its intracellular domain interacts with cytoskeletal proteins like hemoglobin, facilitating cell signal transduction (Figure 2).

### 2.1. Lu/BCAM Can Serve as a Receptor for Laminin

Laminins are matrix proteins that are present in the extracellular matrix and are important components of basement membranes [17]. They are large glycoproteins composed of α, β and γ chains. Currently, five types of α chains, four types of β chains and three types of γ chains have been identified [18]. Laminins can regulate various cellular processes, such as cell adhesion, proliferation, motility, apoptosis and differentiation, by binding to integrin molecules or other receptors [18,19,20,21]. Among the laminins, the α5 chain is a major type in basement membranes [22]. Initial research has shown that Lu/BCAM on the red blood cell (RBC) membrane can interact with laminin α5 [13]. Further studies suggest that the first to third immunoglobulin superfamily domains near the N-terminus of Lu/BCAM are necessary for the interaction between Lu/BCAM and laminin α5 chains [23]. However, there are also reports suggesting the involvement of the fifth domain in laminin α5 binding [24].

The interaction between Lu/BCAM and laminin is essential for the cell adhesion of some tumor and blood cells. Abnormal expression of Lu/BCAM has been observed in certain tumors and sickle red blood cells, which can potentially impact the development of tumors and SCA by influencing the adhesion between cells and laminin [25].

The interaction between Lu/BCAM and laminin also plays an important role in regulating cell migration. The basement membrane protein laminin (LM)-511, which consists of α5, β1 and γ1 chains, is a crucial component of adult basement membranes, and its receptor molecules are primarily integrin α3β1, α6β1 and α6β4 [19,26,27]. Research has demonstrated that in tumor cells, Lu/BCAM competes with integrin α3β1 to bind to LM-511, resulting in weakened intercellular adhesion and an enhanced tumor cell migration ability [14].

### 2.2. Lu/BCAM Can Interact with Integrin

Integrins, as a vital group of cell adhesion molecules, play a crucial role in facilitating cellular attachment to the extracellular matrix. They are involved in regulating various physiological and pathological processes, including vascular development, tumor invasion and metastasis [18,28]. Integrins are comprised of α and β chains, with eighteen α chain subtypes and nine β chain subtypes identified so far. In SCA, integrins in sickle-shaped red blood cells interact with VCAM-1, fibronectin or thrombospondin, thereby regulating blood cell adhesion to the vascular wall and reducing blood flow velocity [29]. An interaction between α4β1 integrin on sickle cell red blood cells and endothelial Lu/BCAM protein has been discovered. This interaction may enhance the adhesion of SS red blood cells to endothelial cells, either by connecting the red blood cells to the endothelial cells or by enhancing the known interactions between α4β1 integrin and other ligands, consequently exacerbating vascular obstructions [27].

### 2.3. Lu/BCAM Can Serve as a Receptor for Cytotoxic Necrosis Factor CNF1

Pathogenic *Escherichia coli* is responsible for over 80% of urethral infections, with its cytotoxic necrosis factor CNF1 playing a significant role in this process [30,31]. CNF1 enters mammalian cells through receptor-mediated endocytosis and contributes to cytoskeletal regulation by continuously activating Rho GTPases through covalent modification [30]. Lu/BCAM has been identified as a key receptor that binds to the C-terminus of CNF1, facilitating its attachment to the cell surface [32,33,34]. And the Ig-like domain 2 of Lu/BCAM was identified as main interaction site by direct protein–protein interaction and competition studies [35]. Thus, Lu/BCAM serves as an essential mediator of CNF1 infection in cells.

### 2.4. Lu/BCAM Can Be Connected to Spectrin

The cytoskeletal protein spectrin, which is crucial for cellular signal transduction, protein transport and cytoskeleton maintenance, interacts with intracellular actin and anchor proteins [36,37,38]. Initially identified in red blood cells, spectrin is now known to be expressed in nearly all mammalian cells [39]. Lu/BCAM can directly bind to spectrin via its C-terminal RK573-574 (Arg573-Lys574) secondary structure near the membrane, and disrupting this binding enhances cell adhesion and spreading mediated by Lu/BCAM through laminin [40,41]. The Lu/BCAM–spectrin connection is implicated in the laminin–actin signaling pathway, promoting stress fiber formation. This process also involves Lu/BCAM–laminin binding and the participation of the RhoA small GTP-binding protein [42,43].

## 3. Regulation of Lu/BCAM

**Figure 3:**
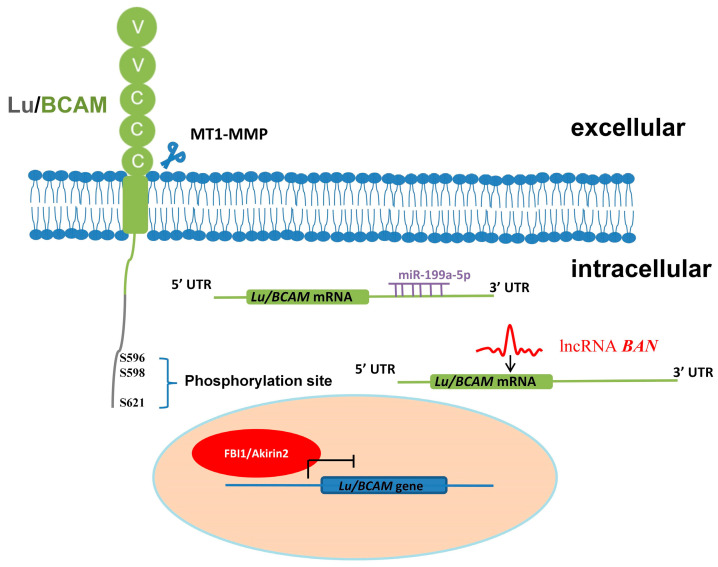
Schematic diagram of regulation of Lu/BCAM. The extracellular membrane domain of Lu/BCAM is susceptible to cleavage by MT1-MMP. Additionally, the intracellular region of Lu/BCAM contains three phosphorylation sites. Lu/BCAM is also targeted by transcription inhibitors FBI1/Akirin2 and miR-199a-5p, which regulate its gene expression. The distribution of Lu/BCAM on the cell surface is regulated by oxidative stress.

Lu/BCAM functions as both a receptor and an adhesion molecule, playing a crucial role in cell signal transduction and cell adhesion. Its expression regulation involves the participation of certain transcription factors, miRNAs or lncRNAs (Figure 3). The phosphorylation modification of Lu/BCAM is regulated by multiple cellular signaling pathways, influencing its adhesion to laminin. Lu/BCAM can be cleaved by matrix metalloproteinases and released into the extracellular space. Additionally, oxidative stress can affect its distribution on the cell surface, thereby influencing its function.

### 3.1. Expression Regulation of Lu/BCAM

The 14-3-3 proteins are a group of phosphoserine/threonine-binding proteins that regulate the activities, localization and interactions of other proteins. The 14-3-3β-binding protein FBI1/Akirin2 sustains ERK1/2 activation by suppressing MKP-1 (MAPK phosphatase) transcription, fostering tumor genesis and metastasis [44]. Studies have revealed that silencing FBI1/Akirin2 expression in rat hepatoma K2 cells leads to a significant upregulation of Lu/BCAM mRNA expression [45]. This suggests that Lu/BCAM may be a target gene regulated by FBI1/Akirin2.

Most miRNAs modulate gene expression by binding to the 3′ untranslated region (UTR) of target mRNA [46]. In keratinocytes, miR-199a-5p shows a negative correlation with Lu/BCAM expression. It directly targets the 3’ UTR region of BCAM mRNA, leading to the downregulation of BCAM expression and consequently, alterations in Lu/BCAM levels [47].

In gastric cancer (GC) cells, a previously unidentified BCAM sense lncRNA, namely BAN, was identified [48]. The knockdown of BAN suppressed BCAM expression at both mRNA and protein levels. Moreover, BAN knockdown inhibited the migration and invasion of GC cells in vitro, but this effect was reversed by BCAM overexpression.

### 3.2. Phosphorylation Modification Regulation of Lu/BCAM

There are five potential phosphorylation sites within the intracellular region of Lu/BCAM. Recent studies have identified sites 596, 598 and 621 as phosphorylation targets. Glycogen synthesis kinase 3 (GSK-3) phosphorylates serine 596 of Lu/BCAM, while serine 598 is phosphorylated by casein kinase II (CKII), and serine 621 is targeted by PKA. The phosphorylation level of Lu/BCAM is intricately linked to its adhesion with laminin [9].

Epinephrine, acting through β2 adrenergic receptors, enhances the adhesion of sickle-shaped red blood cells to laminin α mediated by Lu/BCAM. The cAMP- and PKA-dependent signaling pathways are crucial in this process [25,49]. The activation of the PKA pathway results in the phosphorylation of serine at the 621 site of Lu, thereby augmenting the adhesion ability of Lu/BCAM to laminin.

In human polycythemia vera RBC with the V617F mutant JAK2, there is an upregulation of Rap1-GTP expression, increased Akt activity, elevated phosphorylation levels of Lu/BCAM and enhanced RBC adhesion to laminin compared to wild-type JAK2 [50,51]. The JAK2V617F mutation activates the EpoR-independent Rap1/Akt signaling pathway, leading to the enhanced phosphorylation of Lu/BCAM and thereby increasing RBCs’ adhesion [50].

Moreover, both the dimerization and phosphorylation of Lu/BCAM are indispensable for promoting cell migration [52]. The migration speed of cells expressing the long isoform Lu is faster compared to those expressing the short isoform BCAM, which forms dimers but lacks phosphorylation. Furthermore, serine 621 of Lu has been pinpointed as the specific phosphorylation site targeted by PKA, playing a crucial role in the migration of Madin-Darby canine kidney (MDCK) cells. Additionally, serine 621 of Lu is the phosphorylation target site of PKA involved in MDCK cell migration.

### 3.3. Distribution Modification of Lu/BCAM

Abnormal adhesion of RBC to laminin has been reported in various pathological conditions, with two mechanisms involving Lu/BCAM phosphorylation and dissociation from the spectrin-based skeleton. However, in a recent study, the oxidation-driven mechanism appears to be responsible for the increased adhesion of HD RBC in the absence of Lu/BCAM phosphorylation [53]. Oxidative stress is involved in the post-translational modification of Lu/BCAM, affecting its cellular surface distribution and cis interactions with glycophorin C, thereby activating its adhesion function in sickled dense RBCs.

### 3.4. Lu/BCAM Can Be Cut by MT1-MMP

Matrix metalloproteinases (MMPs) are a class of endopeptidases that rely on metal ions such as Ca^2+^ or Zn^2+^ to primarily degrade the extracellular matrix [54]. Among them, MT1-MMP not only degrades the extracellular matrix but also some adhesion molecules [55,56,57]. In human epidermoid carcinoma cell line A431, the extracellular region near the membrane of Lu/BCAM is cleaved by MT1-MMP, releasing sBCAM into the extracellular space. Low MT1-MMP expression can increase Lu/BCAM expression on the cell membrane [55].

### 3.5. Activation of Lu/BCAM

The aging of red blood cells involves several characteristic changes before their removal from circulation. These changes include the loss of hydration, shedding of membrane, decreased deformability, exposure of phosphatidylserine, decreased sialic acid content and activation of adhesion molecules, such as inducing Lu/BCAM-mediated binding to laminin-α5 [58]. Recent studies show that red blood cell aging is driven by Ca^2+^-dependent K^+^ efflux, known as the Gardos effect [59,60]. Increased intracellular Ca^2+^ activates the Gardos channel, leading to the shedding of vesicles containing glycophorin C (GPC). This results in the loss of deformability and a decrease in the sialic acid content on the membrane. GPC-derived sialic acid inhibits the activity of Lu/BCAM and CD44 by forming complexes on the red blood cell membrane [61]. Lu/BCAM contains a domain that shares similarity with the SIGLEC family of receptors, which are known to bind to sialic acid [62]. In its cis configuration, this domain interacts with sialic acid on erythroid GPC, preventing Lu/BCAM from binding to laminin-α5 in a trans configuration. However, this interaction gradually decreases as erythrocytes age, ultimately resulting in the activation of Lu/BCAM. However, GPC shedding mediated by the Gardos channel activates Lu/BCAM and CD44, causing an increased binding to laminin-α5 and hyaluronic acid.

## 4. Lu/BCAM in Human Diseases

Increasing evidence suggests that Lu/BCAM regulates critical biological processes, such as cell adhesion and migration. Its dysregulation is observed across various hematological diseases and tumors, including lung, liver, colorectal and ovarian cancers, contributing significantly to their pathogenesis. Lu/BCAM holds promise as a molecular marker for clinical diagnosis and as a potential therapeutic target.

### 4.1. Lu/BCAM and Hematological Diseases

#### 4.1.1. Polycythemia Vera

Polycythemia vera is strongly associated with the development of mesenteric and cerebral thrombosis. In this condition, an abundance of blood cells congregate to form erythroid colonies, often accompanied by a V617F mutation in the JAK2 gene [49,50,51]. Within JAK2V617F-mutated red blood cells, the expression and phosphorylation of Lu/BCAM is elevated, enhancing the adhesion between red blood cells and laminin on endothelial cells mediated by phosphorylated Lu/BCAM [50,63,64]. Moreover, research has shown that hydroxyurea enhances the expression and phosphorylation of Lu/BCAM and intensifies the adhesion between red blood cells and the ligand laminin-α5 of polycythemia vera patients [65].

#### 4.1.2. SCA

SCA is a blood disorder caused by mutations in hemoglobin, resulting in the characteristic sickle-shaped red blood cells. In this disease, there is an increased expression of Lu/BCAM in red blood cells, leading to enhanced adhesion between these cells and laminin [66,67]. This, in turn, affects the adhesion between red blood cells and the vascular walls, potentially causing a reduced blood flow velocity [13]. Vascular obstructions, a major acute complication of sickle cell disease, occur due to abnormal adhesion between blood cells and endothelial cells in the microcirculation, which involves integrin α4β1 and its interaction with Lu/BCAM in endothelial cells [27]. VCAM-1, an adhesion molecule expressed on activated endothelial cells, exhibits a high affinity for integrin α4β1. In both dormant and activated endothelial cells, Lu/BCAM is expressed and promotes abnormal adhesion between sickle-shaped red blood cells and dormant endothelial cells. Hydroxyurea can attenuate the adhesion between red blood cells and endothelial cells by activating PDE4A and inhibiting the phosphorylation levels of Lu/BCAM in endothelial cells [68]. Piezo1, a mechanosensitive protein that regulates calcium influx, has been found to increase the exposure of phosphatidylserine on the RBC surface. Piezo1 activation induced the calcium-dependent adhesion of sickle RBCs to laminin, mediated by an increased BCAM binding affinity [69]. Moreover, this mechanism is dependent on the binding affinity of BCAM rather than the expression of BCAM itself.

#### 4.1.3. Severe Malarial Anemia

Severe malarial anemia (SMA) contributes significantly to infant mortality in malaria-endemic regions. The accumulation of uninfected red blood cells (uRBCs) in the spleen is a key factor in SMA. Recent studies have identified the activation of adhesion molecules, including Lutheran/basal cell adhesion molecule (Lu/BCAM) and CD44, on uRBCs from both in vitro Plasmodium falciparum cultures and malaria patients [70]. This activation leads to the binding of uRBCs to specific components of the spleen’s extracellular matrix (ECM), laminin-α5 and hyaluronic acid (HA), respectively. The strong interaction between the ECM and adhesion molecules results in increased calcium levels within uRBCs, an enhanced release of microvesicles and the clustering of Lu/BCAM on altered uRBCs. Ultimately, the activation of Lu/BCAM and CD44 facilitates the binding of uRBCs to ECM components in the red pulp, leading to increased uRBC retention in the spleen. These findings provide insights into a novel mechanism, driven by adhesion molecules, that exacerbates malaria-induced anemia.

#### 4.1.4. HIV-Related Atherosclerotic Lesions

Atherosclerotic lesions are formed by the infiltration of macrophages and low-density lipoprotein cholesterol into the intima of blood vessels. Research has shown that HIV-infected individuals and those with AIDS are at a higher risk of developing peripheral arterial atherosclerosis compared to healthy individuals. Lu/BCAM plays an important role in HIV-related atherosclerotic lesions [71]. The Lutheran blood group antigen Lu^b^ has been associated with a three-fold increase in the risk of HIV infection [72]. This increased risk is believed to be linked to the adhesive properties of Lu^b^, which promote the migration of infected monocytes across endothelial cells and the spread of the virus to distant sites in the body. A recent study showed that the impact of carrying the Lu^b^ antigen as an adhesion molecule on circulating monocytes and neutrophils, as well as its relationship with cholesterol levels [71]. The findings suggest that HIV affects the interaction between monocytes and cholesterol and that this interaction is further influenced by the expression of the Lu^b^ antigen.

### 4.2. Lu/BCAM and Tumors

#### 4.2.1. Skin Tumor

In skin tumor tissues, such as basal cell carcinoma, squamous cell carcinoma and keratoacanthoma, BCAM is upregulated compared to normal skin tissue [73,74]. Within keratinocytes, BCAM predominantly localizes to areas of cell–cell contact, and its expression is correlated with the keratinocyte activation status, potentially influencing processes like cell–cell interactions and migration.

#### 4.2.2. Liver Cancer

The role of Lu/BCAM in liver cancer remains controversial. Lu/BCAM is found to be upregulated in liver cancer tissues, including poorly and well-differentiated tumors, and interacts with integrin α6β1 to competitively bind laminin, modulating the adhesion capacity of liver cancer cells. This involvement contributes to the progression of liver cancer [75]. However, some researchers propose that Lu/BCAM acts as a tumor suppressor gene in liver cancer. FBI1/Akirin2, a binding partner of 14-3-3β and typically associated with tumor promotion, has been found to target Lu/BCAM as a gene in rat liver cancer cells. Silencing FBI1/Akirin2 expression leads to increased Lu/BCAM expression, while the overexpression of Lu/BCAM inhibits crucial biological behaviors, such as clone formation, cell migration and invasion in rat liver cancer cells [44]. 

#### 4.2.3. Bladder Cancer

The regulation of adhesion, migration and metastasis of tumor cells plays a crucial role in the development of cancer [76,77,78,79]. Mutations in Ras genes, such as the H-rasV12 mutant, are known to contribute to cell proliferation, mobility and tumor progression in various human cancers [80]. The mutant form H-*ras*^V12^ can transcriptionally upregulate the expression of Lu/BCAM in bladder cancer cells [16]. The activation of Lu/BCAM-overexpressing cells by its ligand, laminin 10/11, further enhances colony formation, anchorage-independent cell growth and adhesion, while decreasing cell migration through the ERK signaling pathway. This process is accompanied by an elevation of RhoA and the suppression of Rac1 activity. This suggests that the laminin stimulation of Lu/BCAM-mediated cell adhesion plays a role in human bladder tumorigenesis.

#### 4.2.4. Colorectal Cancer

Lu/BCAM and laminin α5 were observed to be upregulated in both tumor cells and the cellular microenvironment of liver metastases in colorectal cancer patients with KRAS mutations [15]. Inhibiting either BCAM or laminin α5 can disturb the adhesion between colorectal cancer cells harboring KRAS mutations and endothelial cells. Therefore, BCAM/laminin α5 may exert a significant role in the metastatic process of colorectal cancer cells with KRAS mutations.

#### 4.2.5. Ovarian Cancer

Advanced serous ovarian cancer, associated with a high mortality rate in women, is typically diagnosed only after metastasis [81]. This tumor type often presents mutations in genes such as TP53, and the genome may undergo extensive gene recombination, resulting in the fusion of multiple oncogenes [82,83]. Studies have reported that among 60 cases of high-grade serous ovarian cancer, 7% exhibit a fusion of BCAM and AKT2 genes. The BCAM–AKT2 fusion gene generates a membrane-associated, constitutively activated AKT2 protein kinase, likely contributing significantly to cancer progression via the PI3K/AKT signaling pathway [84]. Moreover, BCAM plays a crucial role in promoting the invasion of metastatic sites by reducing the compaction of tumor cell spheroids. This effect is achieved by blocking the interaction between laminin α5 and integrin β1 (ITGB1) [11]. BCAM can mediate this function as a membrane-bound protein or after shedding (sBCAM) through the action of ADAM10 or 17.

#### 4.2.6. Gastric Cancer

Elevated levels of BCAM were observed in gastric cancer (GC) tissues with metastasis, compared to those in tissues without metastasis [48]. This upregulation of BCAM was associated with a shorter survival time. The inhibition or knockout of BCAM reduced GC cell migration, invasion and metastasis. Additionally, a previously unidentified BCAM sense lncRNA, known as BAN, was identified. The knockdown of BAN suppressed BCAM expression at both mRNA and protein levels, leading to inhibited GC cell migration and invasion. Clinical data further revealed that increased BAN expression was associated with GC metastasis and a poor prognosis, with a significant correlation to BCAM expression in GC tissues.

#### 4.2.7. Thyroid Cancer

There have been reports suggesting that Lu/BCAM may act as a tumor suppressor gene in certain types of tumors. In thyroid cancer tumor tissue, Lu/BCAM expression is downregulated compared to normal thyroid tissue, and its expression is negatively correlated with tumor size and lymph node metastasis [85].

## 5. Potential Clinical Applications of Lu/BCAM

### 5.1. Biomarkers

Early detection of liver cancer is critical due to its often-asymptomatic nature in the early stages, leading to diagnosis at advanced stages. Detecting liver cancer early is crucial for improving patient survival rates. Lu/BCAM can be cleaved by MT1-MMP1, releasing it from the cell surface into the extracellular space [55,86]. Research indicates that Lu/BCAM is detected not only in liver cancer cell culture media and the blood of liver cancer-bearing mice but also in the blood of liver cancer patients. In mice with liver cancer, the Lu/BCAM concentration in the blood correlates with tumor size, decreasing significantly after tumor removal in liver cancer patients [86]. Additionally, Lu/BCAM is upregulated in the blood of patients with pancreatic and breast cancer [87,88]. These findings suggest its potential as a molecular marker for liver cancer and other tumors, but its presence in the blood of early-stage tumors requires further clinical investigation.

### 5.2. Therapeutic Targets

In colorectal cancer, mutations in downstream molecules of EGFR, such as KRAS, NRAS or PI3K, can lead to a loss of response to anti-tumor drugs targeting EGFR and result in a poor prognosis for patients [89,90,91]. Unfortunately, these patients currently lack effective treatment options. Recent studies have shown that Lu/BCAM is involved in regulating the adhesion between KRAS-mutated colorectal cancer cells and endothelial cells. Inhibiting the function of Lu/BCAM can weaken this cell-to-cell adhesion [15]. This suggests that Lu/BCAM may play a significant role in the metastasis of KRAS-mutated colorectal cancer cells and could potentially serve as a therapeutic target for patients with KRAS mutations in colorectal cancer. Further research is needed to explore the therapeutic potential of targeting Lu/BCAM in this specific patient population.

## 6. Conclusions

In conclusion, Lu/BCAM exhibits widespread expression across blood cells, endothelial cells and epithelial cells of various tissues, primarily functioning as receptors and adhesion molecules. Distributed on the cell membrane, Lu/BCAM binds to ligands or cytoskeletal proteins, facilitating intracellular signal transduction, cytoskeletal maintenance and involvement in biological processes such as cell adhesion and migration. Lu/BCAM was found to be involved in various diseases, including hematological disorders and tumors, suggesting its potential as a potential diagnostic molecular marker or therapeutic target.

## Data Availability

All data are included in the publication.
